# Single-dose tolerability and pharmacokinetics of leritrelvir in Chinese patients with hepatic impairment and healthy matched controls

**DOI:** 10.1128/aac.01377-24

**Published:** 2024-12-17

**Authors:** Cuiyun Li, Jiajia Mai, Min Wu, Hong Zhang, Xiaojiao Li, Haijun Li, Youyun Li, Yanhua Ding

**Affiliations:** 1 Phase I Clinical Trial Unit, First Hospital, Jilin University 12510 , Changchun, China; 2 Department of Anatomy and Neurobiology, School of Basic Medical Sciences, Central South University 618101 , Changsha, China; 3 Guangdong Raynovent Biotech Co. Ltd, Guangzhou, China; Providence Portland Medical Center, Portland, Oregon, USA

**Keywords:** leritrelvir, pharmacokinetics, safety, hepatic impairment, COVID-19

## Abstract

This study evaluated the safety and pharmacokinetics (PK) of a single dose of leritrelvir, a novel inhibitor of 3-chymotrypsin-like cysteine protease (3CLpro), in patients with hepatic impairment versus healthy participants with normal hepatic function. Eight participants with mild (Child–Pugh A) hepatic impairment, eight with moderate (Child–Pugh B) hepatic impairment, and eight healthy matched control participants were enrolled in this open-label, parallel clinical trial. After administration of leritrelvir of 400 mg, PK parameters were calculated and compared across groups. In total, 24 participants were enrolled and completed the study. Leritrelvir was generally well tolerated, with no serious adverse events or deaths reported during the study. Compared to the group with normal hepatic function, the geometric least-squares mean ratios (90% confidence intervals) for *C*_max_, AUC_0-*t*_, and AUC_0-∞_ of leritrelvir in participants with mild hepatic impairment were 96.9% (69.3%, 135%), 92.2% (69.6%, 122%), and 92.1% (69.7%, 122%), respectively. For moderate hepatic impairment, the corresponding ratios were 91.6% (61.7%, 136%), 113% (80.0%, 160%), and 113% (80.0%, 159%). Leritrelvir exposures were comparable among the three groups. Overall, there was no clinically relevant difference in leritrelvir exposure in participants with hepatic impairment compared to normal controls. No dose adjustment is required for leritrelvir in patients with mild or moderate hepatic impairment.

**CLINICAL TRIALS:**

This study is registered with ClinicalTrials.gov as NCT06161259.

## INTRODUCTION

Severe acute respiratory syndrome coronavirus 2 (SARS-CoV-2) has become established in the global population through the coronavirus disease 2019 (COVID-19) pandemic followed by the tragic loss of lives, lockdowns, and social and economic consequences since 2020, and the virus is likely to remain in circulation (1). The rapid development of COVID-19 vaccines proved efficient in saving lives (2). However, potentially pathogenic SARS-CoV-2 variants trigger immune escape and higher infectivity (3). SARS-CoV-2 Omicron variants with highly mutated spike proteins quickly emerged (4). Rapid emergence of highly mutated variants presented new challenges to vaccines and therapeutic antibodies (5). To combat SARS-CoV-2 immune escape, various small-molecule SARS-CoV-2 therapeutics have been developed (6). Of note, nirmatrelvir is the first main protease (Mpro, 3CL) inhibitor of the SARS-CoV-2 and also a cytochrome P450 (CYP) 3A4 substrate (7). Paxlovid is a combination of nirmatrelvir and ritonavir. However, the clinical use of Paxlovid is limited due to drug–drug interactions of ritonavir, which may cause unexpected side effects.

Leritrelvir, a broad-spectrum 3CLpro inhibitor of SARS-CoV-2, was developed by Guangdong Raynovent Biotechnology Co., Ltd. Preclinical evaluation of leritrelvir shows its comparable antiviral activities against SARS-CoV-2 variants with nirmatrelvir. Its improved pharmacokinetics in mice and rats supported its use without ritonavir, which is co-administered with nirmatrelvir (8).

Leritrelvir has a favorable tolerability, safety, and pharmacokinetic proﬁle in the phase 1 study. After a single dose of 200–1,600 mg, leritrelvir was rapidly absorbed, and the maximum concentration (*C*_max_) was reached at 1.3–1.5 h. The average half-life (*t*_1/2_) of leritrelvir was 3–10 h (data not published). Leritrelvir was widely distributed after oral administration, and its protein-binding rate ranged from 74.5% to 79.7%. Leritrelvir was mainly metabolized in the liver by cytochrome P450 (CYP) 3A4 and excreted through feces as the parent drug. In the following phase 2 study, it has been found that leritrelvir monotherapy and leritrelvir plus ritonavir both have clinical potential for the treatment of SARS-CoV-2 infection if administrated within 5 days of the initial positive nucleic acid test result, and their antiviral efﬁcacies are comparable (9).

Considering the above results, it is recommended to use leritrelvir alone to further verify its efﬁcacy and safety in phase 3 study. Leritrelvir monotherapy (400 mg three times a day for 5 days) has good efficacy for mild-to-moderate COVID-19 and does not have serious safety concerns. The primary adverse reactions of leritrelvir were mild hypertriglyceridemia, hyperlipidemia, hyperuricemia, which were tolerable (10). Leritrelvir has been approved as a single-component drug for COVID-19 treatment in China.

Leritrelvir will also be used in patients with mild or moderate hepatic impairment as the patients are at increased risk of COVID-19 (11). Hepatic impairment might modify drug metabolism, reduce drug elimination, and influence drug safety through hepatic blood flow, plasma protein binding, and biliary excretion alterations (12, 13).

Given the involvement of CYP3A in the metabolism/elimination of leritrelvir, it is important to investigate whether impaired hepatic function may impact the pharmacokinetics, safety, and efficacy of leritrelvir. Moreover, it is important to investigate whether leritrelvir dosing modifications are needed for patients with hepatic impairment. According to the Chinese regulatory requirement and US Food and Drug Administration guidance for pharmacokinetic studies in patients with impaired hepatic function, this study was to determine the safety and pharmacokinetics (PK) profile of leritrelvir in patients with mild or moderate hepatic impairment compared with healthy controls (14, 15).

## MATERIALS AND METHODS

### Study population

Eligible men and women aged 18–70 years with a body mass index between 18 and 32 kg/m^2^ were enrolled.

Eligible participants with hepatic impairment should have a confirmed clinical diagnosis of either mild (Child–Pugh A) or moderate (Child–Pugh B) hepatic impairment. Participants with hepatic impairment resulting from primary liver disease, including viral hepatitis type B and C, or non-alcoholic steatohepatitis were eligible. Participants receiving concomitant drugs must have been on stable doses and regimens for at least 4 weeks before screening. Participants with severe hepatic impairment (Child–Pugh C) or aspartate transaminase or alanine transaminase levels >5× the upper limit of normal were excluded.

Healthy participants with normal hepatic function were enrolled in this study. The health status was determined by medical history, physical examination, laboratory tests, abdominal ultrasonography, and electrocardiography. The mean body weight and age of the normal controls were restrained within ±10 kg and ±10 years, respectively, of the mean body weight and age of participants with hepatic impairment. Attempts were also made to ensure comparable male-to-female ratios among the groups.

Subjects who participated in any clinical study within 3 months prior to screening and those with serious infections, trauma, gastrointestinal surgery, or other major surgical procedures within 4 weeks prior to screening were excluded.

This was an open-label, parallel clinical study conducted in a single center in China from September 2023 to October 2023 in accordance with the recommendations of Good Clinical Practice. Participants received a single oral administration of leritrelvir 400 mg.

### Study design

This phase I single-dose, open-label, parallel study investigated the effect of hepatic impairment on the pharmacokinetics and tolerability of leritrelvir. Participants with mild (*n* = 8) or moderate hepatic impairment (*n* = 8) and matched controls (*n* = 8) were enrolled.

Participants were hospitalized and orally administered leritrelvir 400 mg on day 1 after an overnight fast for at least 10 h. During the study period, vital signs and a 12-lead electrocardiogram were used to monitor safety. After safety assessments, participants were discharged on day 4.

### Pharmacokinetic assessments

Blood samples (4 mL) were obtained in collection tubes containing the anticoagulant K_2_-ethylenediaminetetraacetic acid for pharmacokinetic analysis at 0 (pre-dose), 0.25, 0.5, 1, 1.5, 2, 3, 4, 6, 8, 12, 24, 48, and 72 h post-dose. Samples were centrifuged for 10 min (2–8°C, 1,700 × *g*) to separate plasma. All samples were stored at −80°C until analysis. The plasma samples were analyzed for leritrelvir using a validated analytical method based on liquid chromatography-tandem mass spectrometry (LC-MS/MS) using human plasma samples. The linearity range for leritrelvir in plasma was 5.00–5,000 ng/mL. The precision rate was ≤6.4%, and accuracy ranged from 2.9% to 7.3%.

### Pharmacokinetic analysis

The pharmacokinetic population set included participants who received the study drug and from whom samples were obtained for pharmacokinetic analyses. Pharmacokinetic parameters, including the primary parameters *C*_max_, area under the curve from time zero to the last measurable concentration (AUC_0-*t*_), and area under the curve from time zero to infinity (AUC_0-∞_) and secondary parameters time to *C*_max_ (*T*_max_), *t*_1/2_, percentage of the area under the curve that has been derived after extrapolation (AUC__%Extrap_), CL/*F*, and apparent volume of distribution (*V*_*z*_/*F*), were calculated with WinNonlin 8.3 (Certara, Princeton, NJ, USA) using non-compartmental analysis, based on the actual sample collection time. *C*_max_ and *T*_max_ are expressed by measured values. AUC_0-*t*_ is calculated by the trapezoidal method. *λ*_*z*_ is the terminal elimination rate constant obtained from the linear end portion of the logarithmic concentration–time curve. It is obtained by the slope of the straight section at the end of the logarithmic concentration–time curve. AUC_0-∞_ = AUC_0-*t*_ + Ct/*λ*_*z*_ (*t* is the sampling time of the last measured blood drug concentration, Ct is the last time the sample concentration could be measured) and *t*_1/2_ = 0.693/*λ*_*z*_.

### Safety assessments

The safety population included participants who received the study drug. The results of vital signs, 12-lead electrocardiograms, laboratory tests (hematology, clinical chemistry, routine coagulation tests, and routine urinalysis), and physical examinations were included in the safety assessment. Adverse events (AEs) were categorized by severity and their relationship to the study drug. The Common Terminology Criteria for Adverse Events (CTCAE v5.0) were used to classify the severity of AEs.

### Statistical analysis

No formal statistical estimates for sample size were conducted as 8–12 participants were sufficient to evaluate the pharmacokinetics of the test drug according to the National Medical Products Administration guidance for the conduct of PK studies. Therefore, eight subjects were allocated to each cohort.

The primary pharmacokinetic parameters including AUC_0-∞_, AUC_0-*t*_, and *C*_max_ of leritrelvir were log-transformed and subjected to analysis of variance which included hepatic function as a fixed effect. The results were presented as geometric least-squares mean with a 90% confidence interval (CI) for them in different liver function states. Statistical analysis was completed using SAS v9.4 (SAS Institute Inc.) software.

## RESULTS

### Participants

In total, 24 of 51 screened participants were enrolled in this study, and all patients completed the study. The demographic characteristics and baseline variables of the participants are summarized in Table 1.

**TABLE 1:** Demographic data of the study subjects^*a*^

Variable	Mild hepatic impairment (*n* = 8)	Moderate hepatic impairment (*n* = 8)	Normal hepatic function (*n* = 8)	Total (*n* = 24)
Age (years), mean ± SD (range)	52.9 ± 8.87 (37–66)	57.0 ± 7.27 (45–70)	50.8 ± 4.23 (45–57)	53.5 ± 7.25 (37–70)
Body weight (kg), mean ± SD (range)	64.9 ± 8.83 (54.8–79.4)	71.3 ± 6.98 (62.4–81.0)	63.8 ± 5.39 (58.4–75.8)	66.7 ± 7.67 (54.8–81.0)
Body height (cm), mean ± SD (range)	159.5 ± 5.13 (152–169)	165 ± 4.07 (159–172)	164 ± 4.47 (155–169)	163 ± 5.11 (152–172)
BMI (kg/m^2^), mean ± SD (range)	25.5 ± 2.70 (21.4–29.9)	26.1 ± 2.89 (23.1–31.4)	23.6 ± 1.94 (21.9–27.6)	25.1 ± 2.65 (21.4–31.4)
Race, *n* (%)				
Han	8 (100%)	7 (87.5%)	7 (87.5%)	22 (91.7%)
Other	0	1 (12.5%)	1 (12.5%)	2 (8.3%)
Sex, *n* (%)				
Male	5 (62.5%)	7 (87.5%)	6 (75.0%)	18 (75.0%)
Female	3 (37.5%)	1 (12.5%)	2 (25.0%)	6 (25.0%)
Child–Pugh score, mean ± SD (range)	5.13 ± 0.350 (5–6)	7.50 ± 0.760 (7–9)	NA	NA

^*a*^ SD, standard deviation; BMI, body mass index.

### Tolerability of leritrelvir

Leritrelvir was generally well tolerated by all participants. No grade 4 or grade 5 AEs were observed in any subject. No AEs resulted in discontinuation or withdrawal.

As presented in Table 2, 14 AEs were reported by 10 participants (41.7%). Thirteen AEs were adverse drug reactions. One patient aged 56, with mild hepatic impairment, experienced one case of grade 3 decrease in white blood cell (WBC) counts. His white blood cell counts decreased from 2.61 × 10^9^ at screening to 1.74 × 10^9^ on day 4 and recovered to baseline on day 11 without any intervention. The patient reported a history of WBC decrease to this level. Therefore, this AE might be related to the patient’s medical history. All of the other AEs were classified as grade 1 or 2. The most frequent AEs were hyperbilirubinemia (4/24), decreased white blood cell counts (3/24), and decreased neutrophil counts (3/24). More AEs were observed in patients with hepatic impairment. As most of the AEs resulted from hematology abnormality, the hematology summary pre-dose and post-dose of leritrelvir is shown in Table 3. Mean white blood cell counts and neutrophil counts of participants in each cohort decreased in the normal range.

**TABLE 2:** Adverse events summary after administration of leritrelvir^*a*^

Adverse events	Mild hepatic impairment (*N* = 8), *n* (%)	Moderate hepatic impairment (*N* = 8), *n* (%)	Normal hepatic function (*N* = 8), *n* (%)	Total (*N* = 24), *n* (%)
Investigations	4 (50%)	5 (62.5%)	1 (12.5%)	10 (41.7%)
Hyperbilirubinemia	2 (25.0%)	2 (25.0%)	0	4 (16.7%)
White blood cell decreased	2 (25.0%)	1 (12.5%)	0	3 (12.5%)
Neutrophil count decreased	3 (37.5%)	0	0	3 (12.5%)
Hyperuricemia	0	1 (12.5%)	1 (12.5%)	2 (8.3%)
Platelet count decreased	0	1 (12.5%)	0	1 (4.2%)
QT prolongation	0	1 (12.5%)	0	1 (4.2%)

^*a*^ *N* = number of subjects analyzed, *n* = number of subjects.

**TABLE 3:** Hematology summary pre-dose and post-dose of leritrelvir^*a*^

Items	Mild hepatic impairment (*N* = 8)	Moderate hepatic impairment (*N* = 8)	Normal hepatic function (*N* = 8)	Total (*N* = 24)
Red blood cells (10^12^/L)				
Baseline	4.77 (0.48)	4.32 (0.60)	4.78 (0.29)	4.62 (0.50)
D4	4.63 (0.30)	4.29 (0.64)	4.82 (0.41)	4.58 (0.50)
Hemoglobin (g/L)				
Baseline	147.25 (14.84)	140.88 (21.87)	148.00 (8.93)	145.38 (15.73)
D4	144.38 (12.20)	139.38 (19.49)	146.75 (9.29)	143.50 (14.04)
White blood cells (10^9^/L)				
Baseline	4.50 (1.69)	3.87 (1.27)	7.01 (1.29)	5.13 (1.95)
D4	3.98 (1.69)	3.35 (0.72)	6.09 (1.08)	4.47 (1.68)
Neutrophil counts (10^9^/L)				
Baseline	2.51 (0.69)	2.91 (1.06)	4.58 (1.08)	3.33 (1.29)
D4	1.91 (0.63)	2.34 (0.48)	3.84 (0.99)	2.69 (1.10)
Lymphocyte counts (10^9^/L)				
Baseline	1.67 (0.96)	0.63 (0.25)	1.89 (0.66)	1.40 (0.87)
D4	1.75 (1.01)	0.69 (0.30)	1.74 (0.52)	1.39 (0.82)
Platelet (10^9^/L)				
Baseline	116.25 (48.16)	63.75 (31.69)	248.13 (70.75)	142.71 (93.88)
D4	116.13 (52.04)	58.88 (27.86)	251.63 (75.78)	142.21 (98.10)

^*a*^ *N* = number of subjects analyzed. Data are expressed as mean (standard deviation).

### Pharmacokinetics of leritrelvir

**Fig 1:**
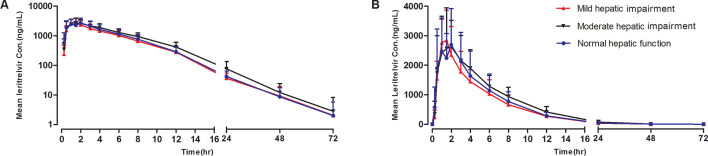
Mean plasma leritrelvir concentrations after administration in participants with normal hepatic function and participants with mild or moderate hepatic impairment (A) semi-log scale and (B) linear scale.

**Fig 2:**
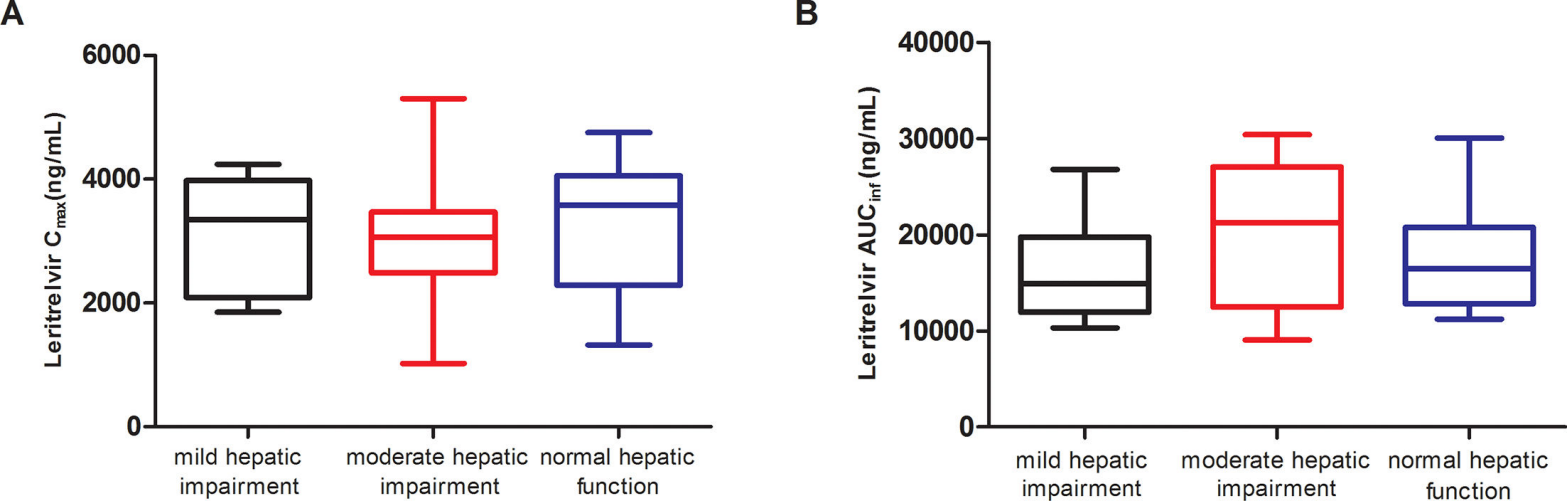
Box plot of leritrelvir *C*_max_ (A) and AUC_inf_ (B) in participants with normal hepatic function and participants with mild or moderate hepatic impairment. AUC_inf_, area under the plasma concentration–time curve from time zero extrapolated to infinity; *C*_max_, maximum observed plasma concentration.

Data collected from the participants were included in the pharmacokinetic and statistical analyses. The mean plasma concentration–time profiles and pharmacokinetic parameters of leritrelvir in participants with normal hepatic function or mild or moderate hepatic impairment are presented in Fig. 1; Tables 4 and 5. The impact of hepatic impairment on leritrelvir exposure is depicted in Fig. 2.

**TABLE 4:** Pharmacokinetic characteristics of leritrelvir^*a*^

PK parameters	Mild hepatic impairment (*N* = 8)	Moderate hepatic impairment (*N* = 8)	Normal hepatic function (*N* = 8)
*T*_max_ (h), median (range)	1.00 (1.00, 1.50)	1.50 (0.500, 3.00)	1.25 (0.500, 3.00)
*C*_max_ (ng/mL)	2,980 (33.9%)	2,820 (49.9%)	3,070 (44.4%)
AUC_0-*t*_ (h · ng/mL)	15,400 (32.6%)	18,900 (48.0%)	16,700 (32.8%)
AUC_0-∞_ (h · ng/mL)	15,600 (32.6%)	19,100 (47.8%)	16,900 (32.4%)
*λ*_z_ (1/h)	0.10 (78.3%)	0.11 (64.8%)	0.10 (84.8%)
*t*_1/2_ (h)	6.83 (78.3%)	6.47 (64.7%)	6.90 (84.9%)
*V*_*z*_/*F* (L)	253 (59.7%)	196 (62.3%)	236 (66.5%)
CL/*F* (L · h^−1^)	25.7 (32.6%)	21.0 (47.8%)	23.7 (32.4%)
MRT_0-*t*_ (h)	5.83 (16.6%)	6.94 (13.9%)	5.92 (13.6%)
MRT_0-∞_ (h)	6.29 (19.0%)	7.32 (15.4%)	6.42 (15.3%)
AUC__%Extrap_ (%)	0.88 (55.3%)	0.63 (85.9%)	0.89 (75.4%)

^*a*^ Data are expressed as geometric means (CV%) unless otherwise specified. MRT, mean residence time.

**TABLE 5:** Statistical analysis of pharmacokinetic parameters of leritrelvir in subjects with varying degrees of hepatic impairment

	PK parameters	Groups	*n*	Geometric least-squares mean	Ratio (%)	90% CI (%)
Mild vs normal	*C*_max_ (ng/mL)	Mild hepatic impairment	8	2,980	96.9	(69.3, 135)
		Normal hepatic function	8	3,070		
	AUC_0-*t*_ (h · ng/mL)	Mild hepatic impairment	8	15,400	92.2	(69.6, 122)
		Normal hepatic function	8	16,700		
	AUC_0-∞_ (h · ng/mL)	Mild hepatic impairment	8	15,600	92.1	(69.7, 122)
		Normal hepatic function	8	16,900		
Moderate vs normal	*C*_max_ (ng/mL)	Moderate hepatic impairment	8	2,820	91.6	(61.7, 136)
		Normal hepatic function	8	3,070		
	AUC_0-*t*_ (h · ng/mL)	Moderate hepatic impairment	8	18,900	113	(80.0, 160)
		Normal hepatic function	8	16,700		
	AUC_0-∞_ (h · ng/mL)	Moderate hepatic impairment	8	19,100	113	(80.0, 159)
		Normal hepatic function	8	16,900		

After a single dose of 400 mg, leritrelvir was rapidly absorbed, and the maximum concentration (*C*_max_) reached at 1.00–1.50 h. Leritrelvir *C*_max_ values were comparable among the three groups. AUC_0-*t*_ and AUC_0-∞_ were ~8% lower in the patients with mild hepatic impairment than those of the normal group. AUC_0-*t*_ and AUC_0-∞_ were ~13% higher in the patients with moderate hepatic impairment than those of the normal group. The 90% CIs for geometric mean ratios of AUC_0-*t*_ and AUC_0-∞_ in mild and moderate groups, all contained unity. Mean *t*_1/2_ was comparable among the three groups (6.47–6.90 h).

**Fig 3:**
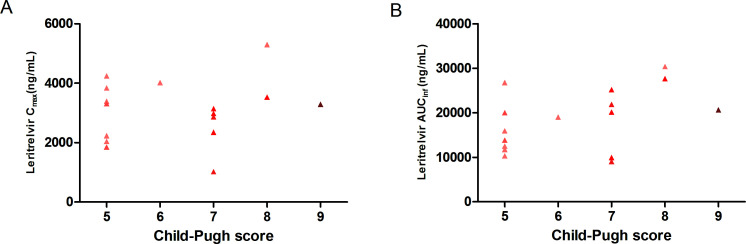
Scatterplots of leritrelvir *C*_max_ (A) and AUC_inf_ (B) versus severity of hepatic impairment (mild [score 5–6] and moderate [score 7–9]). AUC_inf_, area under the plasma concentration–time curve from time zero extrapolated to infinity; *C*_max_, maximum observed plasma concentration.

The Child–Pugh score was not significantly correlated with AUC_0-*t*_ (Spearman’s rank correlation coefficient = 0.109, *P* = 0.687; Fig. 3A) and *C*_max_ (Spearman’s rank correlation coefficient = 0.448, *P* = 0.0817; Fig. 3B).

## DISCUSSION

In this phase 1, single-dose, open-label, parallel study, the tolerability and pharmacokinetics of leritrelvir were assessed in participants with mild or moderate hepatic impairment, as hepatic elimination appears to be the primary route of leritrelvir clearance. Patients with severe hepatic impairment were not enrolled due to safety considerations in this population. One group of participants with normal hepatic functions was enrolled to match the demographics of the patients. No accumulation of leritrelvir was observed in the multiple-ascending-dose study which supported the use of single-dose study. A single dose of 400 mg was chosen based on the dosage in phase 3 study. As plasma protein-binding rate of leritrelvir was lower than 80%, free concentration of leritrelvir was not evaluated in this study.

Overall, a single leritrelvir dose of 400 mg was generally well tolerated by all the participants. More AEs were observed in patients with hepatic impairment. However, no new AEs were observed in this study compared with previous phase 1–3 studies. Most reported AEs were considered mild or moderate in intensity and recovered without intervention. The most common AEs in patients with hepatic impairment were hyperbilirubinemia, decreased white blood cell counts, and decreased neutrophil counts. Though mean white blood cell counts and neutrophil counts of participants in each cohort decreased, they were in the normal range. White blood cell counts decreased with neutrophil counts. In addition, the rates of decreased neutrophil counts in placebo and leritrelvir cohorts were 2.1% and 1.3% in phase 3 study, respectively.

Geometric mean leritrelvir AUC_0-*t*_ and AUC_0-∞_ estimates were comparable among the three groups demonstrating that changes in leritrelvir exposure in patients with hepatic impairment are not clinically meaningful. No dose adjustment is required for leritrelvir in patients with hepatic impairment. Additionally, high inter-subject variability in leritrelvir exposure was observed among the three groups (32.6%–49.9%), which was consistent with previous data in healthy individuals receiving a single 400 mg dose. Given the small sample size of this study, this high inter-subject variability potentially impacted the reliability of pharmacokinetic parameter estimates. Mean *t*_1/2_ was comparable among the three groups.

This study enrolled 11 participants with hepatic impairment who had received concomitant drugs for at least 4 weeks before screening as treatment for hepatitis B virus (HBV) infection or type 2 diabetes mellitus. As all of the concomitant drugs in this study are not inhibitors or inducers of CYP3A4 metabolic enzymes, these drugs would not affect the pharmacokinetic characteristics and safety profiles of leritrelvir.

A completed radiolabeled mass balance study demonstrated that leritrelvir is primarily excreted in feces, either as the parent drug or its metabolite (>90% of the administered dose), indicating that hepatic is the primary organ responsible for its elimination. Hepatic impairment can influence drug pharmacokinetics by altering metabolic enzyme activity, plasma protein binding, and hepatic blood flow, which may affect leritrelvir’s PK profile. In this study, mean albumin concentrations in healthy participants, patients with mild hepatic impairment, and patients with moderate hepatic impairment were 44.9 g/L, 44.6 g/L, and 37.3 g/L, respectively. Since approximately 75% of leritrelvir binds to albumin *in vivo*, the slight decrease in albumin levels in the moderate impairment group is unlikely to have a significant impact on leritrelvir’s pharmacokinetics. Besides, the degree of PK changes depends on both the drug’s properties and the severity of hepatic impairment (16). The 3CL inhibitor GST-HG171, like leritrelvir, is primarily metabolized via hepatic pathways. However, mild to moderate liver impairment does not appear to significantly affect the PK of either drug (17). Similarly, voxelotor, another drug primarily eliminated via hepatic pathways, shows increased exposure only with severe liver impairment, while mild to moderate liver impairment has minimal impact on its PK profile (18). In this study, subjects with mild to moderate hepatic impairment showed no notable effects on leritrelvir’s PK. However, the effect of severe hepatic impairment on leritrelvir remains unknown.

This study was associated with several limitations. First, subjects with severe hepatic impairment (Child–Pugh C) were not included, and only Chinese participants were enrolled. Second, the small sample size may restrict the generalizability of the findings. Further studies with a larger number of participants are warranted to support our results.

### Conclusions

Similar exposures of leritrelvir were observed in participants with mild or moderate hepatic impairment compared with normal controls. A single dose of 400 mg leritrelvir was well tolerated in patients. Therefore, dose adjustment is not considered necessary for patients with mild or moderate hepatic impairment.
